# Model Structure of Human APOBEC3G

**DOI:** 10.1371/journal.pone.0000378

**Published:** 2007-04-18

**Authors:** Kun-Lin Zhang, Bastien Mangeat, Millan Ortiz, Vincent Zoete, Didier Trono, Amalio Telenti, Olivier Michielin

**Affiliations:** 1 Institute of Microbiology, University Hospital Center, University of Lausanne, Lausanne, Switzerland; 2 Life Sciences, Ecole Polytechnique Fédérale de Lausanne, Lausanne, Switzerland; 3 Swiss Institute of Bioinformatics, Lausanne, Switzerland; Duke University Medical Center, United States of America

## Abstract

**Background:**

APOBEC3G (apolipoprotein B mRNA-editing enzyme, catalytic polypeptide-like 3G) has antiretroviral activity associated with the hypermutation of viral DNA through cytosine deamination. APOBEC3G has two cytosine deaminase (CDA) domains; the catalytically inactive amino-terminal domain of APOBEC3G (N-CDA) carries the Vif interaction domain. There is no 3-D structure of APOBEC3G solved by X-ray or nuclear magnetic resonance.

**Methodology/Principal Findings:**

We predicted the structure of human APOBEC3G based on the crystal structure of APOBEC2. To assess the model structure, we evaluated 48 mutants of APOBEC3G N-CDA that identify novel variants altering ΔVif HIV-1 infectivity and packaging of APOBEC3G. Results indicated that the key residue D128 is exposed at the surface of the model, with a negative local electrostatic potential. Mutation D128K changes the sign of that local potential. In addition, two novel functionally relevant residues that result in defective APOBEC3G encapsidation, R122 and W127, cluster at the surface.

**Conclusions/Significance:**

The structure model identifies a cluster of residues important for packaging of APOBEC3G into virions, and may serve to guide functional analysis of APOBEC3G.

## Introduction

Primate APOBEC3G has antiretroviral activity associated with the hypermutation of viral DNA through cytosine deamination (for recent review see [1]–[3]). Human APOBEC3G (huAPOBEC3G) fails to restrict HIV-1 due to the degradation imposed by the HIV-1 Vif [4]. In contrast, a number of primate APOBEC3G orthologs display activity against HIV-1 [5]–[8]. APOBEC3G has a duplicated catalytic deaminase domain (CDA); the amino-terminal domain (N-CDA) of APOBEC3G is required for viral encapsidation but not cytosine deamination [9]–[11].

There is no 3-D structure solved by X-ray or NMR nor an accurate model of APOBEC3G available. APOBEC3G relates to APOBEC family and AID (activation-induced deaminase) at the sequence level. Recent comparative modeling work for APOBEC-1 and AID [12]–[17] led to the proposition of a secondary structure alignment between APOBEC3G and cytidine deaminase [18]. The recent publishing of the crystal structure of APOBEC2 [19] provides the template to build a reliable model structure of APOBEC3G by theoretical methods.

In the present work, we model human APOBEC3G, with particular emphasis on the N-terminal domain. Using mutant data of the N-CDA, we mapped critical residues for packaging of APOBEC3G into viral particles on the new structure model of the huAPOBEC3G N-CDA. We completed the analysis by mapping N-CDA residues that are under positive evolutionary pressure in primate APOBEC3G.

While this work was concluded, Huthoff and Malim provided a detailed molecular genetic analysis of the N-CDA region spanning amino acids residues 119 to 146 (Ref [20]). This analysis defined residues 124 to 127 as having a role in APOBEC3G packaging into HIV-1 virions, and residues 128 to 130 as crucial for the interaction with HIV-1 Vif. Our current results confirm and extend this work, and place the findings in a detailed structural model that may serve to advance rational drug design.

## Materials and Methods

### Target sequence and template for structural model

The huAPOBEC3G sequence (residues 1-384; NCBI accession: NP_068594, see http://www.ncbi.nih.gov/) was defined as target sequence. The newly crystallized Human APOBEC2 dimer [19] (PDB ID 2NYT obtained from Xiaojiang S. Chen, see http://www.rcsb.org/pdb/Welcome.do) served as template.

### Target-template alignment

We used the align2d function of MODELLER program (http://salilab.org/modeller/) [21]–[23], to align huAPOBEC3G N-CDA sequence (residues 1-194) and huAPOBEC3G C-CDA sequence (residues 195-384) to the huAPOBEC2 dimer. Additional assessment of the target-template alignment compared structurally determined (DSSP, http://bioweb.pasteur.fr/seqanal/interfaces/dssp-simple.html) [24] and predicted (PSIPRED, http://bioinf.cs.ucl.ac.uk/psipred/) [25]–[27] secondary structures. The alignment was analyzed and viewed by Jalview (http://www.jalview.org/) [28].

### Model building, evaluation and mapping of key residues

The target-template alignment was used to build the model by satisfaction of spatial restraints. The ANOLEA program (http://swissmodel.expasy.org/anolea/) [29]–[31], that estimates the folding free energy of each residue of a protein chain to assess the quality of the predicted structure, was used to score all the models, using the default 5 residue window averaging.

The geometry of the active site/pseudo-active site was based on the corresponding homologous active site regions of the huAPOBEC2 template. The main interaction of anti-parallel β2-β2′, between huAPOBEC3G N-CDA and C-CDA was defined by restraining segments 48-56 and 235-243 as anti-parallel β-sheet, based on the huAPOBEC2 dimer. For the chain connection between N-CDA and C-CDA, the first 20 amino acid residues of C-CDA were used as a linker and modeled ab initio using the loop routine of the MODELER program.

Generation of 100 models allowed selection of the best model candidate based on the global ANOLEA score. Final refinement for alignment of gap regions was performed by generating 100 additional models, followed by selection of the best model on the basis of the ANOLEA score. The final model was energy-minimized using the CHARMM program (http://www.charmm.org/) [32] and the CHARMM22 all atom force field [33]. The minimization consisted of 200 steps of steepest descent using a dielectric constant of 1 and the Generalized Born GB-MV2 implicit solvent model without cutoff for the solvation free energy. Model evaluation was based on ANOLEA with window 5 averaging, as described above.

The CHARMM program was used to calculate the solvent accessible surface area of the final model. Additionally, using the FoldX program (http://foldx.embl.de/) [34], in silico alanine-scanning was performed by mutating each residue and calculated the change of folding energy. Molecular surface visualization was done using Chimera (http://www.cgl.ucsf.edu/chimera/) [35], [36]. The surface was color-coded according to the Poisson-Boltzmann potential calculated by the UHBD program [37].

### Mutation analysis, constructs, viral production and titration

The plasmid expressing a hemagglutinin (HA)-tagged form of APOBEC3G was a kind gift from M. Malim. A series of APOBEC3G alanine and specific mutants was constructed with the QuickChange Mutagenesis kit (Stratagene). The collection was already constituted, and it was not defined on the basis of the new structural data. HIV-1 particles were produced by transient transfection of 293T cells with Fugene (Roche) of a wild-type or of a Vif-defective HIV-1 proviral clone. Viral titers were determined in single-round infectivity assays by applying filtered supernatant from producer cells on HeLa-CD4-LTRLacZ indicator cells. Virion infectivity was derived by dividing the infectious titer by the amount of physical particles.

### Packaging assay and Western Blots

HIV-1 particles were produced by transient transfection of 293T cells with Fugene (Roche). 1 ml of virus was then spun in Eppendorf tubes at 13'000rpm in a microfuge at 4° for 90 minutes, without sucrose cushion. Pellets were resuspended in PBS 1% Triton, and the virion amount was measured by a standard RT assay. Normalized amount of virions were then loaded on standard Laemmli protein gels to perform Western Blots. Cell extracts were obtained through a standard RIPA extraction procedure. The HA tag was detected with the mouse hrp-coupled anti-HA 3F10 antibody (Roche). PCNA (proliferating cell nuclear antigen) was detected with the mouse Ab-1 antibody (Oncogene Science). The HIV-1 capsid was detected with the murine anti-p24 antibody produced from the AIDS Research and Reference Reagent Program #183-H12-5C.

## Results

**Figure 1:**
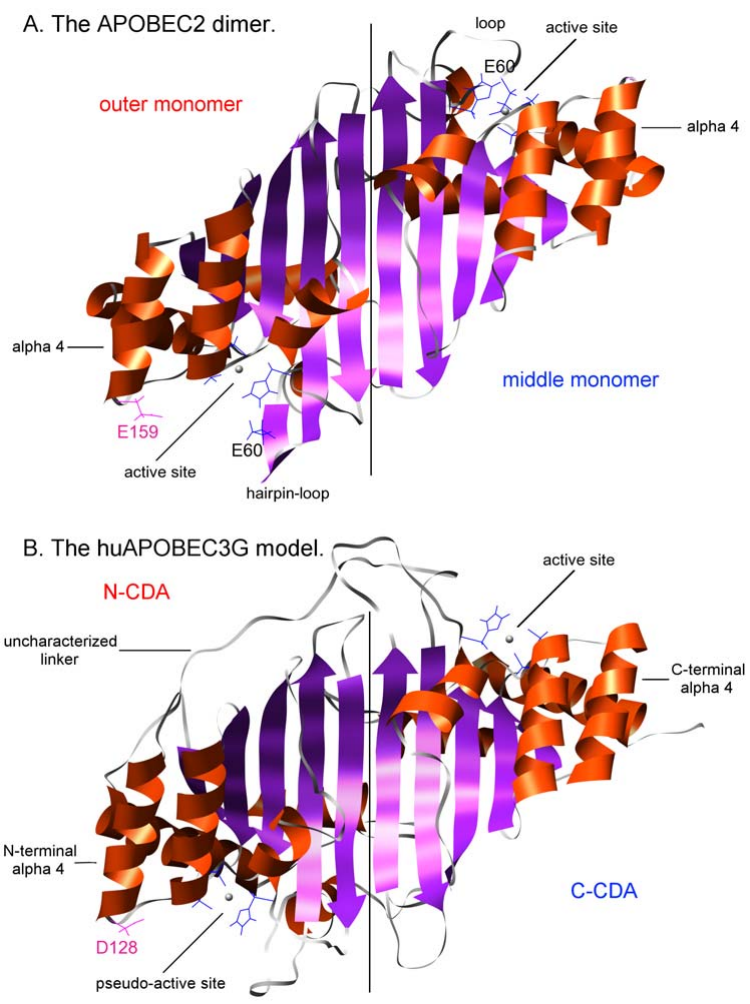
Ribbon view of the huAPOBEC2 dimer and the model of huAPOBEC3G. Panel A. The APOBEC2 “homo”-dimer. Panel B. The huAPOBEC3G model underscoring the six active and pseudo-active site residues in blue, the two zinc ions, and the position of the residue D128, key in the interaction with HIV-1 Vif.

The huAPOBEC2 tetramer is composed of two outer monomers and two middle monomers. The outer and middle monomers share the same sequence but a slightly different structure in the region of residues 57-68. For an outer monomer, this region is a hairpin-loop; but for a middle monomer this region is a loop with residue E60 coordinating with Zn^2+^ [19], **Fig. 1A**. We modeled the huAPOBEC3G N-CDA based on the outer monomer of APOBEC2 to resolve the structure between residues 22 and 33 of huAPOBEC3G. The outer monomer of APOBEC2 is more suitable as template because the middle monomer coordinates the Zn^2+^ with E60, a residue not present at the corresponding position in huAPOBEC3G N-CDA. The corresponding region of huAPOBEC3G C-CDA was modeled ab initio. The target-template alignment generated by MODELLER agreed with the secondary structure alignment (Supplementary Fig. S1A). The resulting huAPOBEC3G model is shown in **Fig. 1B**. The structural pattern of this model is very similar to that of the huAPOBEC2 dimer. The final model has a good ANOLEA score profile (indicating reliability of the structure prediction) with a pattern comparable to that of huAPOBEC2 dimer despite the low sequence identity (27%), **Supplementary Fig. S1**.

### Mutation analysis and mapping of key functional residues

Mutation analysis interrogated 48 of 194 (25%) residues of the huAPOBEC3G N-CDA **(Table 1)**. Mutation of the conserved residues constituting the N-CDA Zn^2+^ coordination domain H65, C97 and C100 prevented or reduced encapsidation as previously reported [9]. Mutation of residues F70 and Y91, shown to mediate RNA binding [38], was associated with reduced APOBEC3G encapsidation, as previously reported [9]. Mutation of the pseudo-catalytic site residue E67 (E67Q), resulted in poor protein expression, which limited functional evaluation; E67A was previously reported to reduce the rates of APOBEC3G encapsidation into HIV1 virions [9]. E67 and F70 are buried inside of the model; E67A and F70A may affect folding stability (in silico alanine-scanning). Y91 is a partially exposed at the surface.

**Table 1:** Mutation, antiviral activity, and surface exposure of residues of the huAPOBEC3G N-CDA.

	Anti-HIV activity*	Protein expression**	Solvent Accessible Surface Area ***
Y13A	+++	+++	14.1+0
F17L	+++	+++	2.4+0.3
F21L	+++	+++	0.4+0
S28A	++	+++	73.9+17.3
R29A	+++	+++	187.2+0
T32A	++	++	6.5+0
Y37A	+++	+++	0+0
K40A	+	+	76.0+0.2
S45A	++	++	33.9+9.5
L49A	+++	+++	24.8+0
L62A	+++	+++	50.1+4.3
H65R	+	+	7.5+0
E67Q	+	+	0+0
F70L	0	++	0.2+0
F74L	++	+++	151.5+15.0
E85Q	+++	+++	104.9+0
Y86A	+++	+++	16.3+0.8
W90L	+++	ND	0+0
Y91A	0	++	16.7+0
I92V	+++	ND	0.1+0
S93A	+++	+++	0+0
W94L	+	+++	35.0+0
P96L	++	++	0+0
C97S	+	+	15.0+1.5
C100S	+	+	0+0.2
M104A	+++	+++	0+0
F107L	+++	+++	23.6+0
L108A	++	++	2.5+0.6
L116A	++	++	0.5+0
T117A	+++	+++	19.1+0
I118A	++	+	0+0
**R122A**	0	++	76.6+0
L123A	++	++	0+1.6
Y124A	+	+++	36.1+0.7
Y125A	+++	+++	138.6+6.4
F126L	+++	+++	22.6+0
**W127L**	0	++	187.3+17.8
D130K/N	+++	+++	88.7+2.1
Y131V	++	++	2.2+0
E133Q	+++	+++	112.3+4.9
L135A	+++	+++	0+0
L138A	++	++	0+0
M152A	++	++	0+0
Y154A	++	+++	120.7+0
F157L	++	+++	4.4+0
C160S	+++	+++	0.2+0
F164L	++	++	0+0

Novel functional residues that abolish huAPOBEC3G antiviral activity against wild-type HIV-1 infection are highlighted bold.

* 0 = no activity, +residual activity (<10% of wild type),++modest activity (10–50%), +++full activity.

** Semiquantitative western blot

*** Estimated by side chain+backbone. Values greater than 20 suggest that the residue is exposed at the surface.

**Figure 2:**
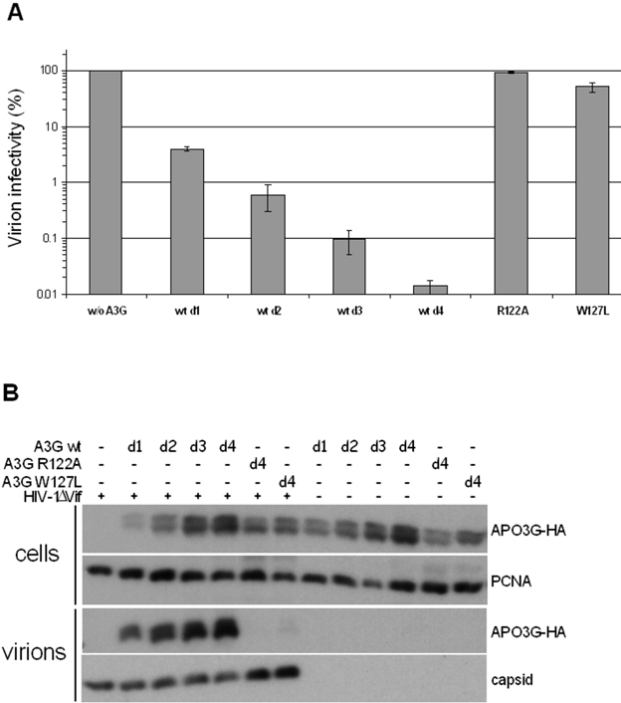
Infectivity, and packaging assay of novel defective huAPOBEC3G mutants. Panel A. Δ Vif HIV-1 particles were produced in presence of different doses of wt APOBEC3G-HA (A3G), or with the highest dose of the R122A and W127L mutants of APOBEC3G-HA. The infectivity of these particles was determined by titration on P4.2 indicator cells. The doses d1 to d4 of APOBEC3G correspond respectively to a molar ratio of APOBEC3G-HA plasmid to virus plasmid of 0.6∶1, 1.3∶1, 2.7∶1 and 5.5∶1. The graph is made from one duplicate experiment, and is representative of a total of at least 4 independent duplicate experiments. Panel B. The presence of wt APOBEC3G-HA or the R122A and W127L mutants in ΔVif HIV-1 virions was assessed by western blot. The PCNA and capsid western blots serve to check for consistent loading of cellular and virion extracts, respectively. Representative of at least two independent experiments.

**Figure 3:**
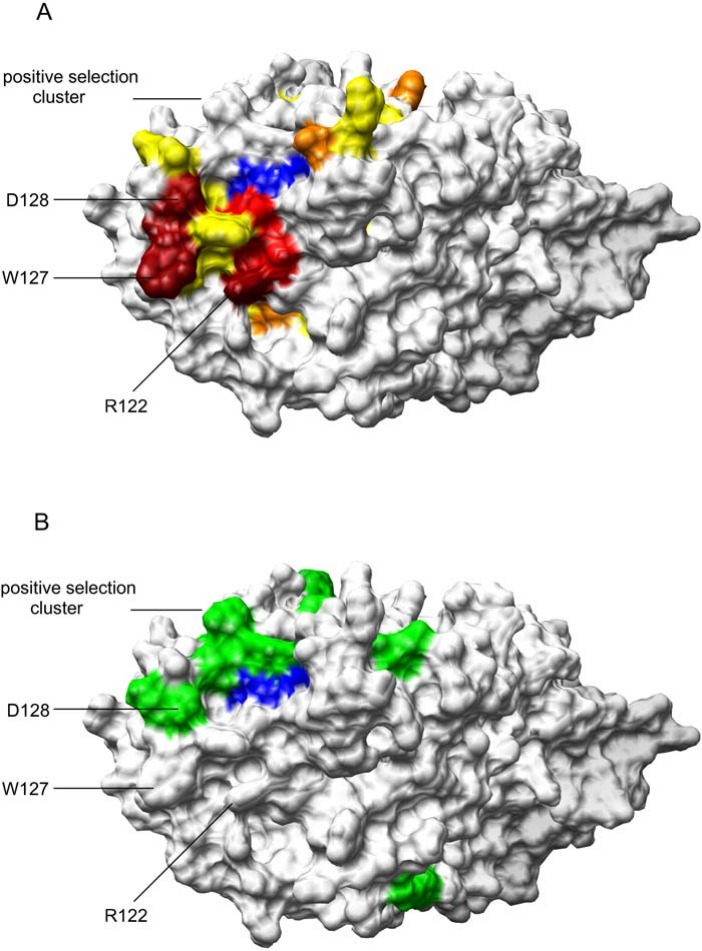
Mapping of functional and evolutionary informative residues at the molecular surface of N-CDA. Panel A, Mapping of functional residues. The color gradient (dark red>red>orange>yellow) reflects the role of various mutations in abolishing anti-HIV activity (see Table 1) ranging from no antiviral activity (dark red) to normal activity (yellow). Panel B. Mapping of evolutionary informative residues at the molecular surface. Green color identifies amino acids under positive selective pressure in primate APOBEC3G. The positive selection cluster includes T98, K99, R102, D128, and P129. Blue color identifies the pseudo–active site.

**Figure 4:**
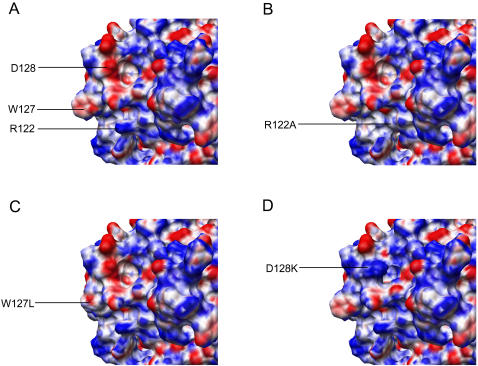
Mapping potential to molecular surface. Zoomed in view on the functional surface region within the N-CDA. Panel A, wild-type huAPOBEC3G N-CDA. Panel B-D, effect of mutations R122A, W127L, and D128K on surface charge and shape. The red color represents negative potential, the blue color expresses positive potential, and the white color expresses zero potential.

Amino acids substitutions at positions Y13, F17, F21, R29, T32, Y37, K40, S45, L49, L62, E85, Y86, W90, I92, S93, P96, M104, F107, L108, L116, T117, I118, L123, Y125, F126, D130, Y131, E133, L135, L138, M152, C160 and F164 did not result in changes in anti-viral activity. Mutations S28A, F74L, W94L, Y124A, Y154A resulted in reduced inhibition of ΔVif HIV1, while mutations R122A and W127L completely abolished this activity (**Table 1**). The functionally defective phenotype of these mutants correlated with a failure to become encapsidated into ΔVif HIV-1 particles (**Fig. 2**). R122, W127 and D128 form a cluster at the surface (**Fig. 3**) and contribute to changes in surface charge or structure (**Fig. 4**).

Prior evolutionary analysis of primate APOBEC3G identified a number of residues under diversifying (positive) selection [39]. Most of the residues under positive selection pressure are exposed at the surface of the model. A cluster of residues under positive selection that includes T98, K99, R102, D128, and P129 overlaps with the cluster of functionally important residues around D128 (**Fig. 3**).

## Discussion

This work presents a model structure of huAPOBEC3G that captures information from the recently published APOBEC2 structure [19]. Critically, the APOBEC2 template provides a structural reference for the predicted extra helix α4, that carries residue D128–a residue that governs the virus-specific sensitivity of APOBEC3G to Vif-mediated inhibition [5]–[8]. Previously available CDA structures provided suboptimal templates for APOBEC3G N-CDA. This is not only due to the low sequence identity, but also because of the presence in APOBEC3G N-CDA of an extra α helix. In contrast, APOBEC2 has an extra α helix, resulting in a α1-β1-β2-α2-β3-α3-β4-α4-β5-α5-α6 configuration, while CDA structures such as human cytidine deaminase have the pattern α1-β1-β2-α2-β3-α3-β4-β5-α4-α5. In addition, the direction of β4 and β5 are different: parallel in APOBEC2 but anti-parallel in cytidine deaminase. We had previously used the yeast Cytosine deaminase (PDB ID 1P6O, chain A) to address the position of the extra helix and the correct direction of β4 and β5.

The model emerging from this analysis allows speculation on various functionally relevant structural details of interest for the understanding of the Vif-APOBEC3G interaction and the process of APOBEC3G encapsidation into HIV-1 virions. In contrast with previous secondary and tertiary structure models, the current model locates the distinctive extra alpha helix on the same planar surface as the pseudo-active site. We evaluated extensive mutation data on this surface, that characterized two functionally relevant residues R122 and W127 that resulted in failure to inhibit infection by HIV-1/Δ*vif* due to lack of packaging of APOBEC3G into viral particles. Interaction of APOBEC3G with the NC-domain of HIV-1 Gag and non-specific RNA binding leads to its encapsidation into progeny virions [10], [40]–[43]. The structural model proposes a surface hot-spot domain that includes residues R122, W127 in proximity to the active site, and overlapping with a cluster of residues under positive selective pressure that includes D128 and P129.

Our results are consistent with the work of Huthoff and Malim [20]. While their work concentrated on the molecular genetic analysis of the 28-amino acid region between residues 119 and 146, our analysis interrogated 48 of the 194 residues of the N-CDA domain. This reflects the interest to identify additional motifs capable of interacting with HIV-1 Vif, or participating in packaging [20], [44]. Minor differences were observed between the two studies. We observed a defect in the packaging efficiency of R122A mutant protein–that extends the relevant motif to include R122-Y124-Y125-F126-W127. Regarding the motif affecting regulation by Vif, our analysis of D130 mutants (D130K and D130N) did not result in a Vif-resistant phenotype. This difference may reflect a greater dependence of the N-CDA and Vif for the negative charge in position 128 (Ref [20]). While not formally tested in the present study, the biological relevance of P129 is highlighted by its inclusion–together with D128-in a patch of residues under positive selective pressure in primates.

Inspection of surface modifications conferred by various mutations highlights the structural and/or charge differences in this region as the molecular basis for disruption of the APOBEC3G packaging into HIV-1 virions, and modification in the interaction with Vif. The detailed model structure presented here could serve to advance rational drug design. The quality of a homology model is strongly related to the sequence identity with the structural template. The current model, based on a sequence identity of 27%, should be satisfactory in its global fold, as supported by the good ANOLEA energy score profile (**Supplementary Fig. S1**). From such a model, correct positioning of the protein backbone and orientation of the side chains can be expected, allowing reliable conclusion to be made in the design and interpretation of experimental mutation data. Whether or not the accuracy of such a model is sufficient to perform docking simulations is still an open question. However, the fact that critical residues obtained experimentally by mutation analysis do cluster in well-defined patches at the surface of the model argue that side chain packing is correct. In the latter case, docking simulations could be envisioned based on the current model. As recently shown for TRIM5α [45], the model proposed may serve to guide further functional analysis of APOBEC3G.

## Supporting Information

Figure S1 Panel A, Target-template alignment. Alpha helix are shown in red and beta sheets in green. The huAPOBEC3G N-CDA is aligned to the outer (O) monomer of APOBEC2, and the C-CDA to the middle (M) monomer. Panel B, ANOLEA scores. The three ANOLEA profiles and the secondary structure were aligned according to the target-template alignment. The ANOLEA profile excludes the uncharacterized linker (residues 195-214)
(2.86 MB EPS)
